# Weight Gain in Children with Cleft Lip and Palate without Use of Palatal Plates

**DOI:** 10.1155/2012/973240

**Published:** 2012-12-06

**Authors:** Renato da Silva Freitas, Andrey Bernardo Lopes-Grego, Helena Luiza Douat Dietrich, Natacha Regina de Moraes Cerchiari, Tabatha Nakakogue, Rita Tonocchi, Juarez Gabardo, Éder David Borges da Silva, Antonio Jorge Forte

**Affiliations:** ^1^Center for Integral Assistance of Cleft Lip and Palate, Federal University of Paraná, Cuitiba, PR, Brazil; ^2^School of Medicine, Yale University, New Haven, CT 06510, USA

## Abstract

*Goals/Background*. To evaluate children's growth in the first year of life, who have cleft palate and lip, without the use of palatal plates. *Materials/Method*. Chart review was conducted, retrospectively, in the Center for Integral Assistance of Cleft Lip and Palate (CAIF), in Brazil, between 2008 and 2009. Results for both genders were compared to the data published by the World Health Organization (WHO) regarding average weight gain in children during their first year of life. *Results*. Patients with syndromic diagnosis and with cleft classified as preforamen were excluded, resulting in a final number of 112 patients: 56 male and 56 female. Similar patterns were seen comparing the two genders. Although it was observed weight gain below the average until the 11th month in male patients and until 9 months in female patients, both genders remained at the 50th percentile (p50) and improved after the 4th month of age for boys and the 9th month of age for girls. *Conclusion*. Children with cleft palate weigh less than regular children during their first months of life. At the end of the first year, weight gain is similar comparing normal and affected children. However, factors that optimized weight gain included choosing the best treatment for each case, proper guidance, and multiprofessional integrated care.

## 1. Introduction

Cleft lip and palate is the most common craniofacial malformation and is often associated with swallowing impairment and decreas growth rate, likely secondary to children's inability to feed appropriately [1]. According to the literature, children with either cleft lip or palate have a short, fast, uncoordinated, and ineffective intraoral suction, which may cause asphyxia, the entrance of milk in the nose cavity, and as excessive air ingestion [2, 3]. The cleft is considered the main anatomic issue leading to these dysfunctions. In fact, studies showed that children with clefts have lower height and weight when compared to a control group, especially during the first year of life [4–6].

On the other hand, Mcheik and Levard report that the growing curves of children with cleft palate, and regular children are equivalent if patients have no syndrome or severe malformations associated [7]. Consequently, the growing pattern might be more influenced by external factors, such as parents' adaptation to the children's condition or the feeding method used [8]. Adjustments on the care of these children may be helpful to assure appropriate nutrition and significant weight gain [9]. 

Therefore, the main priority during the first months of life, including those with cleft palate, should be appropriate nutrition. This is especially important to those who are candidates for future surgery. Healthy weight in children is not only related to good response to infections or surgery stress but also to adequate recovery during the postoperative period [9]. 

Different feeding techniques were described for children with clefts to improve nutritional status. They vary from breastfeeding and special dietary supplements to spoons and modified syringe use [10]. However, the best choice is the one that the family and the child learn from providers who work on reference centers [7]. One technique, intending to increase the suction of these patients, is the use of palatal plates. Palatal plates intend to facilitate suction for these patients. Some authors also report that plates contribute to remodel the palatal arch, helping intraoral suction during the first months of life [11]. However, in our center, we do not use palatal plates. Therefore, our study evaluates the growth of children with cleft palate and cleft lip in their first year of life, without the use of palatal plates.

## 2. Methods

This study was approved by the Ethics in Research Committee of the Hospital de Clínicas from the Federal University of Paraná in Brazil, registration number: Banpesq 2012025932. Medical records from patients treated in the Center for Integral Assistance of Cleft Lip and Palate (CAIF), in the city of Curitiba, Brazil, during the period from 2008 to 2009 were carefully analyzed. All new cases of cleft lip, with cleft palate associated and cleft palate isolated from patients under one year of age, were included in this study. The data was obtained retrospectively.

The following data points were considered: (1) patient's name, gender, and birth date; (2) cleft classification; (3) type of milk consumed: human breast milk, cows' milk, or formula; (4) time length of exclusive breastfeeding; (5) age of solid food consumption; (6) weight at birth; (7) monthly weight during the first year of life.

The results found for boys and girls were then compared to the data published by the World Health Organization (WHO) regarding average weight gain in children during their first year of life. 

## 3. Results

135 patients were included in this study. Patients with syndromic diagnosis and with cleft classified as preforamen were excluded, resulting in a final number of 112 patients: 56 male and 56 female.

Regarding food type, the most prevalent was the use of nutritional formulas alone (36.6% of the sample), followed by the combined use of breast milk and formula (25%). Exclusive use of breast and cow's milk were 8.9% and 2.7%, respectively. The average duration of exclusive breastfeeding was 46 days, and the average age for initiation of solid foods was 8 months.

**Figure 1:**
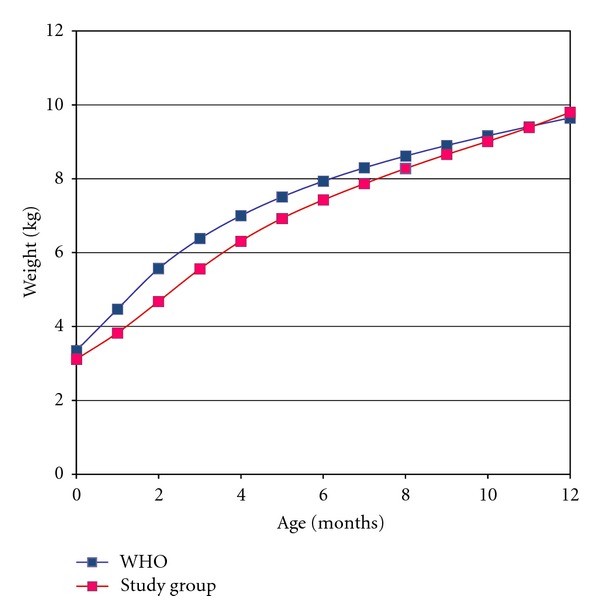
Comparison between the average weight gain of boys with cleft lip and palate estimated by WHO. Blue dots: WHO; pink dots: study group.

Interestingly, a similar weight gain pattern was seen on both genders.  Figure 1 shows that the weight gain in affected male patients is below the average for their age; by the 11th month, on the other hand, the curves were almost superimposed. At birth, the boy's weight is about 200 g below the average for this age. This difference increases as time goes by, reaching almost 900 g in the third month of life; however, after the third month, the difference between the average weight gain begins to decrease. In fact, around the tenth month, affected infants weight gain exhibit a similar pattern to normal.

**Figure 2:**
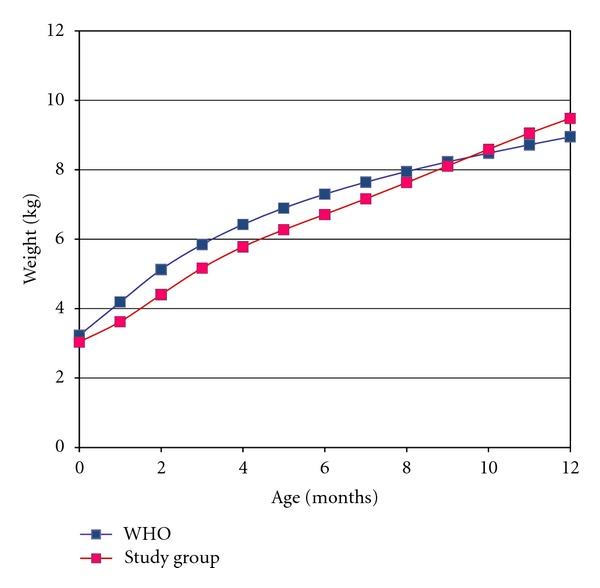
Comparison between the average weight gain of girls with cleft lip and palate estimated by WHO. Blue dots: WHO; pink dots: study group.

Figure 2 represents the weight gain of female patients compared with normal values. Girls weight gain shows a difference of almost 200 g compared to the expected weight gain. There is a gradual increase of this discrepancy up to the third month of life, when it peaks at almost 750 g. Afterwards, the gap diminishes reaching a similar pattern near the ninth month of life.

**Figure 3:**
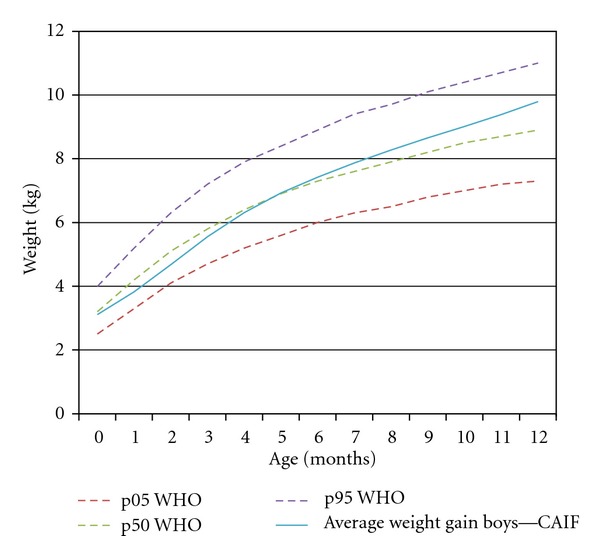
Curve of weight gain for boys compared to percentiles estimated by WHO.

**Figure 4:**
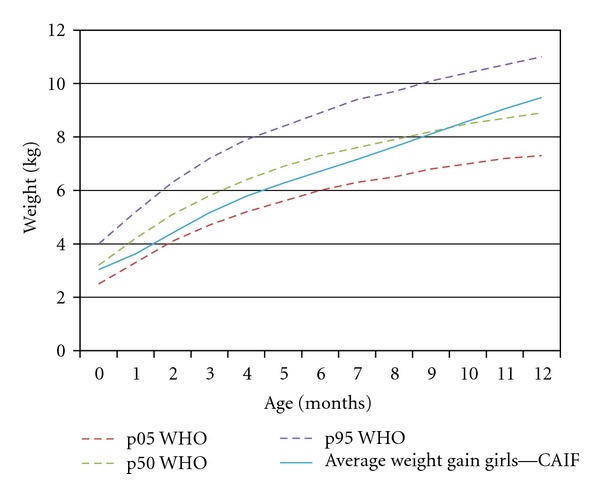
Curve of weight gain for girls compared to percentiles estimated by WHO.

 Weight gain curve in both genders, demonstrated in Figures 3 and 4, is plotted near the 50th percentile (p50) line for each time of assessment, crossing it after the 4th month of age for boys and 9th months of age for girls.

## 4. Discussion

Children with clefts have a higher rate of malnutrition secondary to suctioning impairment. This was first presented by Fabricius and Aquapendente in 1916 [7]. Since then, multiple discussions about the actual interference of clefts in growth rate and weight gain have taken place [9].

Weight gain and growing becomes an issue during the first month of life, probably due to their difficulty to generate negative intraoral pressure, necessary to accomplish a good suction [12]. As a result, the process becomes quick, inefficient, and not coordinated with the swelling movements, that can result in insufficient absorption of nutrients and deficiency in weight gain [13]. In this context, the majority of children require some mechanical support to facilitate the flow of milk [10]. However, the most effective solution to this problem is the palate corrective surgery, which occurs between 9 and 12 months in most centers. The use of devices that facilitate suction and allow a greater intake of breast milk, such as the palatal plate, is an alternative protocol followed by some services [14, 15]. These centers promote the benefits and advantages of palatal plate use. Consequently, some families and pediatricians question the choice of not using it. Trying to shed some light into this discussion, a randomized study compared the weight gain and growth of children who used and did not use the prosthesis, finding no statistically significant difference in the variables evaluated. For that reason, the research did not recommend the universal use of the palatal plate [15]. 

 Another aspect widely discussed is the role played by different nutritional formulas and the use of breast milk on the growth pattern and weight gain in infants with cleft palate [16]. In our study, we did not identify any association between the milk type and the weight gain measured. Our study noticed a predominant use of nutritional formulas, despite the policy of supporting breastfeeding adopted by our hospital. This is probably due to the intrinsic difficulty to collect breast milk and the convenience of formula use.

Affected patients experience an obvious growth deficit in the early months of life in both genders. It is possible that this lower weight gain during this period is a consequence of cleft infant's adaptation to new methods of nutrition [17]. It is important to emphasize that, even though the initial weight gain is lower than the one established by WHO (Figures 1 and 2), the sample data is near to p50, especially the boys (Figures 3 and 4). Breastfeeding is often hampered by the lack of knowledge of parents or caregivers about the proper ways of feeding the cleft child and difficult access to appropriate treatment centers for education and care.

After patients' initial access to health care professionals, it was noted improvement in growth and weight gain, indicating that affected infants, when appropriately stimulated to reach a targeted nutritional level, can achieve normal infant's growth. Therefore, proper orientation and access to followup are factors that contribute to improve patient's nutritional status [18]. Weight gain greater than WHO's mean percentile after 10 months of age reaffirms the success of the current feeding guidelines, follow-up regimen and treatment. 

## 5. Conclusion

In our study, none of our cleft palate patients used palatal plates, and both genders weighed less than regular infants during their first months of life, in comparison to the curves established by the World Health Organization. However, at the end of the first year, they presented similar weight gain in both groups. In addition, we noticed that the type of cleft palate and the type of milk consumed had no influence neither on weight gain nor on growth. Therefore, the factors that improved weight gain were: choosing the best treatment for each case, support, education, integrated multiprofessional care and regular following up. Parents' collaboration, working together with health professionals, allowed adequate growing of cleft palate children, minimizing health risks.
